# Bronchopulmonary Penetration of Isavuconazole in Pulmonary Transplant Recipients (PBISA01): Protocol for a Phase IV Clinical Trial With a Single Treatment Arm

**DOI:** 10.2196/37275

**Published:** 2022-09-14

**Authors:** Ignacio Darnaude-Ximénez, Antonio F Caballero-Bermejo, Belén Ruiz-Antorán, Myriam Aguilar-Pérez, Alicia Gómez-López, Aranzazu Sancho-López, Cristian García-Fadul, Elena Diago-Sempere, Manuel Valle Falcones, Piedad Ussetti-Gil, Cristina Avendaño-Solá, PBISA01-Study Group

**Affiliations:** 1 Clinical Pharmacology Department Hospital Universitario Puerta de Hierro-Majadahonda Instituto de Investigación Sanitaria Puerta de Hierro-Segovia de Arana Madrid Spain; 2 Pneumology Department Hospital Universitario Puerta de Hierro-Majadahonda Instituto de Investigación Sanitaria Puerta de Hierro-Segovia de Arana Madrid Spain; 3 Mycology Reference and Research Laboratory Centro Nacional de Microbiología Instituto de Salud Carlos III Majadahonda Spain; 4 Hospital Universitario Puerta de Hierro-Majadahonda Instituto de Investigación Sanitaria Puerta de Hierro-Segovia de Arana Madrid Spain

**Keywords:** isavuconazole, triazoles, invasive fungal infections, fungus, fungal, infection, pharmacokinetic, bronchoalveolar lavage, bronchoalveolar lavage fluid, bronchoscopy, lung transplant, bronchopulmonary level, bronchopulmonary, epithelial lining fluid, bronchiole, alveolar macrophage, pharmaceutical, drug efficacy

## Abstract

**Background:**

Aspergillosis is the most frequently observed invasive fungal disease (IFD) in lung transplant recipients. Isavuconazole (ISA) has shown a better safety profile and noninferiority to voriconazole in the treatment of patients with IFD.

**Objective:**

The aim of this study is to describe the bronchopulmonary pharmacokinetic profile of oral ISA by analyzing the degree of penetration in the epithelial lining fluid and alveolar macrophages in patients receiving lung transplantation with a diagnosis of IFD.

**Methods:**

A total of 12 patients aged ≥18 years receiving a lung transplant with an IFD diagnosis and indication for ISA treatment and follow-up bronchoscopy will be included in the study. After 5 days of treatment with ISA and before the treatment is discontinued, the patients will be randomized (1:1:1:1) to perform the scheduled bronchoscopy at various times after the administration of ISA (2, 4, 8, and 12 hours). In total, 4 blood samples will be obtained per patient: at 72 hours after treatment initiation, on the day of the bronchoscopy, at the time of the bronchoalveolar lavage (simultaneously), and at 7 days after treatment initiation, to analyze tacrolimus and ISA plasma levels. ISA concentrations will be measured in plasma, epithelial lining fluid, and alveolar macrophages by a high-performance liquid chromatography/UV coupled to fluorescence method.

**Results:**

Enrollment for the PBISA01 trial began in October 2020 and was completed in October 2021. All samples will be analyzed once recruitment is complete, and the results are expected to be published in October 2022.

**Conclusions:**

There are no clinical studies that analyze the bronchopulmonary penetration of ISA. Bronchoalveolar lavage performed routinely in the follow-up of lung transplant recipients constitutes an opportunity to analyze the bronchopulmonary penetration of ISA.

**Trial Registration:**

European Clinical Trials Register 2019-004240-30; www.clinicaltrialsregister.eu/ctr-search/trial/2019-004240-30/ES

**International Registered Report Identifier (IRRID):**

DERR1-10.2196/37275

## Introduction

### Background

Invasive fungal diseases (IFDs) are a frequent cause of morbidity and mortality in solid-organ transplant recipients. Lung transplant recipients are especially vulnerable to the development of these infections as a result of the peculiarities of the pulmonary graft, permanent contact with the external environment, and the high levels of immunosuppression necessary for the prevention of rejection [1]. Aspergillosis is the most frequently observed IFD in lung transplant recipients [2]. Despite the universal prophylaxis with voriconazole and nebulized amphotericin B, a nonnegligible percentage of patients present with *Aspergillus* infection [3,4]. The period of greatest risk is immediately posttransplant. However, the risk is maintained throughout the posttransplant evolution.

Triazoles are effective drugs against *Aspergillus* infection. Voriconazole is the drug of first choice for the treatment of invasive aspergillosis [5,6]. However, it is not free of adverse effects.

Isavuconazole (ISA) is indicated in adults for the treatment of invasive aspergillosis and mucormycosis in patients for whom amphotericin B is inappropriate. ISA has shown a better safety profile and noninferiority to voriconazole in the treatment of patients with IFD [7] and is being increasingly used. Data from healthy volunteers demonstrated high oral bioavailability, high hepatic metabolism, and an extended elimination half-life [8].

The efficacy of ISA depends on fungal exposure to the drug and the minimum inhibitory concentration of the microorganism. To achieve the desired therapeutic effect in lung infections, it is necessary to ensure that the drug penetrates properly at the bronchopulmonary level. As it has already been shown for other azoles [9-11], the properties of ISA will greatly determine its distribution to tissues, organs, compartments, and fluids [12]. There are no clinical studies that analyze the bronchopulmonary penetration of ISA.

In this sense, the bronchoalveolar lavage (BAL) performed routinely in the follow-up of lung transplant recipients constitutes an opportunity to analyze the bronchopulmonary penetration of ISA. The simultaneous determination of the levels of ISA in plasma and the epithelial lining fluid (ELF) obtained through the BAL can be useful to determine its penetration at the bronchopulmonary level and correlate it with the response to treatment [13]. This aspect is especially relevant as lung transplant recipients are a population that is especially susceptible to the development of IFD.

### Goal of the Study

The aim of this study is to describe the pharmacokinetic profile of oral ISA at the bronchopulmonary level through the degree of penetration in the ELF in patients receiving lung transplantation with a diagnosis of IFD.

## Methods

### Design

This is a single-center, phase IV, prospective, noncontrolled, open-label, and single-arm clinical trial aimed at describing the pharmacokinetic profile of oral ISA at the bronchopulmonary level. This will be done through analyzing the degree of penetration in the ELF from lung transplant recipients diagnosed with IFD. A total of 12 patients aged ≥18 years receiving a lung transplant with an IFD diagnosis and indication for ISA treatment according to the Summary of Product Characteristics will be included in the study.

**Figure 1:**
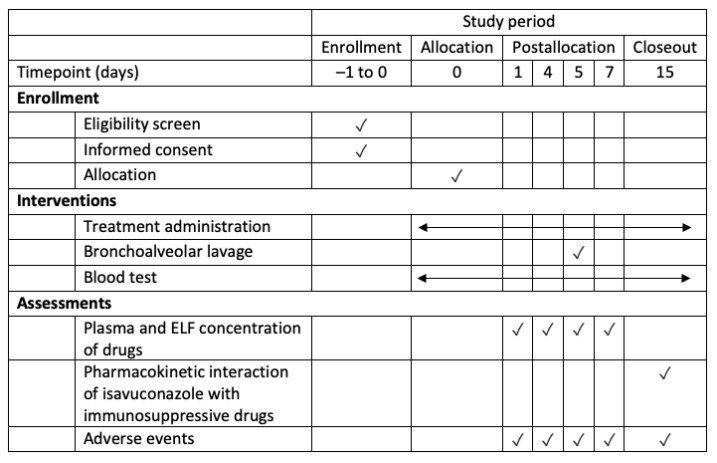
Schedule of enrollment, interventions, and assessments. ELF: epithelial lining fluid.

This clinical trial has been registered on the European Clinical Trials Register (2019-004240-30). This study will adhere to the SPIRIT (Standard Protocol Items: Recommendations for Interventional Trials) 2013 statement (see Figure 1 for the SPIRIT figure of enrollment, interventions, and assessments and Multimedia Appendix 1 for the SPIRIT checklist).

### Ethics Approval

This study will be carried out in accordance with the principles emanating from the Declaration of Helsinki (2013) and the Oviedo Convention of the Council of Europe of 1997, ratified in 1999, and according to current legal regulations in Spain (Royal Decree 1090/2015 and EU Clinical Trials Regulation 536/2014).

The project, the final amended protocol (version 1; October 31, 2019), and the consent form have been reviewed and approved by the Research Ethics Committee at Hospital Universitario Puerta de Hierro-Majadahonda (approval 167/19) and the Spanish Regulatory Authority (Spanish Agency of Medicines and Medical Devices).

Eligible patients may only participate in the study after providing written informed consent approved by the Research Ethics Committee.

Informed consent must be obtained before performing any specific study procedure. The process of obtaining informed consent should be documented in the patient’s source documents (medical history).

### Participants and Recruitment

The patients will consecutively be enrolled from October 2020 to June 2022 in Hospital Universitario Puerta de Hierro-Majadahonda. All the patients will be hospitalized.

Before enrollment, participants will be provided with detailed information about the clinical study, including its purpose, processing, scheduling, and possible risks and benefits. All patients will be required to sign an informed written consent before any study procedures commence.

### Sample Size

In total, the inclusion of 12 lung transplant recipients is planned. Currently, there are no data in the scientific literature about the pulmonary disposition of ISA. The number of subjects has been estimated based on the objective and characteristics of the study and the drug under study. Given the descriptive nature of the study and previous similar studies [9,10], it has been considered that with the exposure of 12 subjects, the precision of the estimates will be sufficient to determine the pharmacokinetic profile of oral ISA at the bronchopulmonary level.

Patients eligible for inclusion in this study must meet all the inclusion criteria and should not meet any of the exclusion criteria (Textbox 1).

**Inclusion and exclusion criteria.**

**Inclusion criteria**

- Recipient of a lung transplant, aged ≥18 years, with an indication of treatment with isavuconazole according to the Summary of Product Characteristics
- No limit between the date of transplant and the date of onset of the fungal infection requiring treatment with isavuconazole
- Intent and ability to follow scheduled visits, treatment plan, laboratory analysis, and other study procedures
- Legally competent and able to understand, sign, and date the informed consent form
- Signing of the written informed consent in accordance with Good Clinical Practice and local legislation, obtained before any study procedure

**Exclusion criteria**

- Allergy or intolerance to isavuconazole
- Contraindication for bronchoscopy or bronchoalveolar lavage
- Any clinical condition or analytical disorder that, in the opinion of the investigator, are considered clinically relevant to participate in the study
- Individuals who show an inability to follow the instructions or collaborate during the study
- Women who are pregnant or undergoing breastfeeding
- Having participated in another clinical trial during the 3 months prior to the start of the study in which a research or commercially available drug was tested
- Lack of intent or inability to follow the procedures described in the protocol
- Inability to grant written informed consent

### Intervention

The trial has been approved as a “low interventional clinical trial” according to the definition in the European Clinical Trials Regulation (536/2014), because the investigational medicinal product is used in accordance with the terms of the marketing authorization, and the additional diagnostic or monitoring procedures do not pose more than minimal additional risk or burden to the safety of the subjects compared to normal clinical practice.

All participants included will receive the product under investigation—oral ISA—at the doses and dosage regimen stated in the Summary of Product Characteristics: loading dose of 200 mg/8 h for the first 48 hours and maintenance dose from day 3 at 200 mg/24 h.

After 5 days of treatment with ISA and before the treatment is discontinued, patients will be randomized to perform the scheduled bronchoscopy at different times from the administration of ISA with the following distribution:

- Group 1 (n=3): 2 hours from the administration of ISA
- Group 2 (n=3): 4 hours from the administration of ISA
- Group 3 (n=3): 8 hours from the administration of ISA
- Group 4 (n=3): 12 hours from the administration of ISA

Furthermore, 4 blood samples will be obtained per patient: at 72 hours after treatment initiation, on the day of the bronchoscopy, at the time of the BAL (simultaneously), and at 7 days after treatment initiation, to analyze tacrolimus and ISA plasma levels.

There is no restriction regarding the use of medications and concomitant therapies. All medications (except study medication) and nondrug therapies administered during the study should be listed in the corresponding section of the concomitant medication case report form (CRF). The use of any concomitant treatment will be carried out according to the authorized conditions of use or, failing that, according to the usual clinical practice.

### Outcome Measures

#### Primary End Point

The primary outcome is the measurement of ISA in the ELF. ISA concentrations will be measured in plasma ELF and alveolar macrophages by a high-performance liquid chromatography/UV coupled to fluorescence method. ISA levels in the ELF and alveolar macrophages will be obtained from the BAL. The proposed chromatographic method will allow the quantification of ISA in the clinical samples after a first step of protein precipitation and the direct injection (plasma) of resulting supernatant. For BAL samples, a previous step of sample concentration will be required to increase the sensitivity of the method. The analytical run will result in the specific characterization and robust quantification of this triazole by its UV and fluorescent profile.

Data from the cell count, percentage of alveolar cells, and volume of cell-lining fluid will be presented for the different times when bronchoscopy is performed. The volume of cell-lining fluid will be estimated using the concentration of urea in plasma and BAL from the following formula [13]: *estimated ELF volume = amount of total urea in BAL (mg) / plasma urea concentration (mg/mL)*. The area under the curve of ISA will be estimated at the ELF level. The concentration, time-dependent curves in the ELF will be calculated using noncompartmental analyses. The ratio between the ELF and plasma will be estimated from the average values of the patients included in each group.

#### Secondary End Points

For interaction evaluation, tacrolimus and mycophenolate concentrations will be determined before treatment and at 72 hours, 96 hours, and 7 days from the initiation of ISA treatment. The percentage of patients who required dose adjustment of the immunosuppressive drug after the start of treatment with ISA and the magnitude of the adjustment will be determined.

Safety will be assessed by recording adverse effects, the physical examination of patients, and analytical results.

A systematic description of all adverse events recorded during the follow-up will be performed. Listings of adverse events, previously coded by the Medical Dictionary for Regulatory Activities and grouped by organ or systems according to severity, intensity, and causal relationship with the study medicinal products, will be included.

### Adverse Events

From the signing of informed consent to the end of the study, all adverse events that the investigator considers related to the study procedures will be recorded.

As part of the medical history, health problems (including clinically significant vital analytical values or constants located outside the reference range) that were diagnosed or known before the signing of informed consent will be recorded.

### Data Management and Quality Control

All records will be collected in a CRF, which will be completed by a trained and qualified investigator. Once a CRF is completed, the original record will not be changed if any corrections are made. The completed CRF will be reviewed by the clinical monitor.

Data entry and management will be guided by medical statistics experts. After reviewing and confirming that the established database is correct, the data will be locked by the main researchers and statistical analysts. The locked data or files will not be changed thereafter and will be submitted for statistical analysis by the research group. The Clinical Trial Unit of Instituto de Investigación Sanitaria Puerta de Hierro-Segovia de Arana, which does not have any competing interests, will be responsible for monitoring the data.

The study will be safely conducted in accordance with the protocol and applicable regulatory requirements under Good Clinical Practice, and data collection will be properly executed.

### Statistical Analysis

The statistical analysis will be carried out following the principles specified in the International Conference on Harmonization Topic E9 (CPMP/ICH/363/96). The data will be analyzed using the SPSS statistical software (version 22.0; IBM Corp).

Given the characteristics of the study—a descriptive study—only 2 analysis groups are planned: (1) the safety and tolerability analysis group, which will include all patients who have received at least 1 dose of ISA; and (2) the pharmacokinetic analysis group, which will include all patients with BAL samples from bronchoscopy.

Demographic data and other data related to patient selection will be descriptively summarized for the pharmacokinetic analysis group and the safety and tolerability group. Categorical data will be presented in the form of frequencies and percentages. For continuous data, descriptive statistics such as mean, median, and SD or median, IQR, minimum, and maximum will be presented.

## Results

The enrollment for the PBISA01 trial began in October 2020 and was completed in October 2021. Thirteen patients have been enrolled in this clinical trial. No data regarding the pharmacokinetic profile of ISA has been analyzed yet. The results are expected to be published in October 2022.

## Discussion

### Expected Findings

There are no clinical studies that analyze the bronchopulmonary penetration of ISA. Our hypothesis is that the bronchopulmonary penetration of ISA in lung transplant recipients may be compromised as a result of poor vascularization and local inflammatory phenomena.

In this sense, the BAL performed routinely in the follow-up of lung transplant recipients constitutes an opportunity to analyze the bronchopulmonary penetration of ISA. The simultaneous determination of the levels of ISA in plasma and the ELF obtained through the BAL can be useful to determine its penetration at the bronchopulmonary level and correlate it with the response to treatment [13]. This aspect is especially relevant as the lung transplant recipients are a population that is especially susceptible to the development of IFD.

The use of BAL samples as surrogates of ELF and alveolar macrophages exposure of ISA requires the development of a sensitive method of analysis. The proposed chromatographic method includes one important advantage: the dual detection of UV and fluorescence. The fluorescence detection results in sensitivities between 1 and 3 orders of magnitude higher than absorption methods.

It is well-accepted that penetration into the site of infection to achieve microbe-eliminating concentrations is a key requirement for the efficacy of all antimicrobial agents [14]. The concentration of ISA within the lung ELF may provide a good estimate of drug exposure for the treatment of IFD [12]. However, the monitoring of ISA concentration in the ELF via bronchoscopy is not practical in the clinical setting, so the use of plasma ISA concentration as a surrogate for the ELF concentration is a good alternative.

The pharmacokinetics of ISA has been studied as an experimental treatment of invasive aspergillosis in neutropenic rats. In one study [15], the subjects treated with ISA had a lower residual fungal load after treatment, lower lung damage, greater survival, and lower levels of (1,3)-β-D-glucan, both in plasma and the BAL.

Other physiological factors such as the presence of inflammation and changes in vascularization substantially influence the exposure of drugs at the site of action. This aspect is especially relevant in the lung transplant recipient in which the absence of bronchial revascularization and local inflammatory phenomena secondary to ischemia and reperfusion can hinder the tissue penetration of drugs.

The design of the study includes the randomization of the patients to perform the scheduled bronchoscopy at different times after dose intake. This is a suitable design to collect ISA pulmonary concentration at different timepoints without interfering with the scheduled dosage and with no additional burden to the patient. The number of subjects has been estimated based on the objective and characteristics of the study and the drug under study. Given the descriptive nature of the study and previous similar studies [9-11], it has been considered that with an exposure of 12 subjects, the precision of the estimates will be sufficient to determine the pharmacokinetic profile of oral ISA at the bronchopulmonary level.

### Conclusions

To the best of our knowledge, this study will be the first well-designed clinical trial to analyze the bronchopulmonary penetration of ISA in a steady-state situation in patients receiving a lung transplant.

## Appendix

Multimedia Appendix 1 SPIRIT (Standard Protocol Items: Recommendations for Interventional Trials) checklist.

## Abbreviations

BAL: bronchoalveolar lavage
CRF: case report form
ELF: epithelial lining fluid
IFD: invasive fungal disease
SPIRIT: Standard Protocol Items: Recommendations for Interventional Trials
